# Development of a Mechanism-Based Next-Generation Therapeutic for Heart Failure Derived From the Dark Genome

**DOI:** 10.1016/j.jacbts.2023.09.006

**Published:** 2023-12-26

**Authors:** Janika Viereck, Thomas Thum

**Affiliations:** a Cardior Pharmaceuticals GmbH, Hannover, Germany; b Institute of Molecular and Translational Therapeutic Strategies (IMTTS), Hannover Medical School, Hannover, Germany

**Keywords:** cardiac remodeling, cardiovascular trials, contractile function, HFpEF, HFrEF, microRNA therapeutics

## Abstract

The ability of nucleic acids for intramolecular interactions opens manifold opportunities for novel medicines that have the potential to treat intractable human disorders, including heart disease. In this context, microRNAs have been identified as pleiotropic regulators of disease pathways and consequently as powerful therapeutic targets. With antisense oligonucleotides novel drug modalities are available to specifically inhibit as well as correct derailed microRNAs including pathological downstream pathways potentially restoring hallmarks of disease. However, only a handful of microRNA-targeting drugs underwent clinical testing so far, and none in the cardiovascular field. In this paper, the authors introduce the first-ever microRNA-based therapy that entered clinical trials in heart disease and present the previous development from target identification to first-in-human studies.

Complementary base-pairing is a very elegant mechanism invented by nature creating the unique structure of the DNA and preserving the code of life. Not least through the discovery of noncoding RNAs, it has become apparent that this principle opens many more possibilities. Base-pairing shapes molecular interactions, like microRNAs that by simple complementary target binding orchestrate numerous biological and disease-related phenomena. Moreover, since the development of antisense oligonucleotides (ASOs), base-pairing emerged as powerful tool to selectively block the activity of transcripts enabling completely new therapeutic strategies, not at least for the treatment of cardiovascular diseases. Combining both, the basic scientific concept of microRNA, 20-nt short endogenous RNAs, as a potential drug target and an ASO as a therapeutic agent has emerged into a first-ever full clinical drug development program (Central Illustration) aiming to address the unmet medical need of heart failure (HF).^1^

**Central Illustration:**
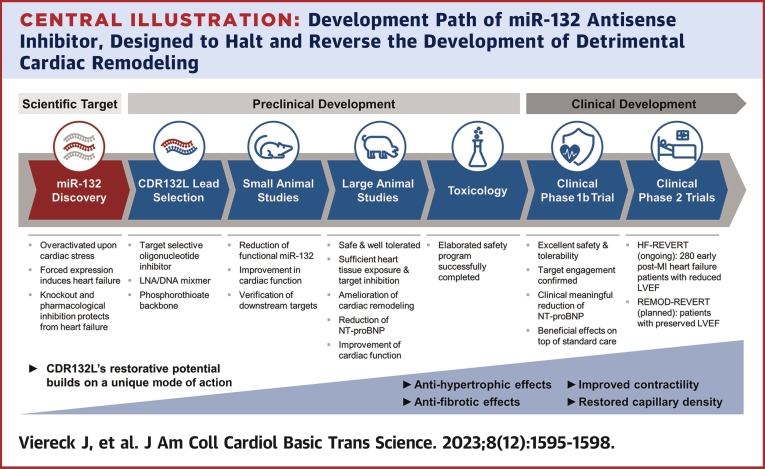
Development Path of miR-132 Antisense Inhibitor, Designed to Halt and Reverse the Development of Detrimental Cardiac Remodeling LNA = locked nucleic acids; LVEF = left-ventricular ejection fraction; MI = myocardial infarction; NT-proBNP = N-terminal prohormone of brain natriuretic peptide.

## A Therapeutic Concept with a Unique Mode of Action

As a potential therapeutic target, the conserved microRNA miR-132 is well documented to be overactivated in the heart upon various cardiac stress^2^ and binds to the 3'-untranslated regions of mRNAs, provoking target degradation and/or translational repression. miR-132 targets multiple genes in parallel (eg, transcription factor FoxO32, calcium transporter, and ATPase SERCA2A3) resulting in a combinatorial effect on related downstream and cardiovascular disease-related signaling cascades, including autophagy, calcium handling, cardiac contraction, hypertrophy, and fibrosis. In the heart, miR-132 overactivation provokes adverse remodeling and contractile dysfunction, which are implicated in the development and progression of HF.^2^, ^3^, ^4^

As a therapeutic strategy, CDR132L was developed as a first-in-class, lead-optimized, synthetic ASO inhibitor directed against miR-132, designed to halt and reverse the development of detrimental cardiac remodeling, and is currently under clinical development. CDR132L is a molecule composed of locked nucleic acid (LNA) and DNA building blocks connected via a phosphorothioate backbone enhancing binding affinity and nuclease-resistance. The drug, formulated in saline solution, is delivered parenterally without any excipients. Once administered into the systemic circulation, the physicochemical properties enable sufficient target tissue uptake and exposure with a long tissue half-life.^2^^,^^3^ In cardiac cells, CDR132L selectively binds to miR-132 and sterically blocks the access of the cellular machinery (RNA-induced silencing complex) that mediates the interaction between the microRNA and its target transcripts. Thus, selective inhibition of aberrant miR-132 by CDR132L results in target de-repression and subsequently normalization of multiple signaling pathways finally reversing ischemic and nonischemic-related adverse cardiac remodeling and restoring normal cellular functions, contributing to improved cardiac performance.^2^, ^3^, ^4^, ^5^

## Fast Forward Path From the Scientific Target to the Clinical Candidate

miR-132 was identified in a discovery approach for miRNAs that drive maladaptive cardiac growth by testing a library of different miRNA precursors in cardiomyocytes. The microRNA was found to be overactivated by different prohypertrophic stimuli in vitro and upon pressure-overload induced cardiac hypertrophy in vivo. Transgenic mice with cardiomyocyte-specific overexpression of the miR-132 cluster developed signs of cardiac remodeling as well as severe HF and died prematurely, whereas mice lacking miR-132 were partially protected from pressure-overload induced HF. In line with the phenotypical observations in gain-of- and loss-of-function studies, pharmacological inhibition of miR-132 rescued cardiac hypertrophy and HF in mice.^2^ Based on this therapeutic concept, CDR132L was rationally designed and selected as lead candidate for in vivo proof of concept and, subsequently, as a candidate for clinical development.

Initially evaluated in a miR-132 transgenic HF mouse model, intravenous (IV) CDR132L treatment reduced functional miR-132 level and cardiac dilatation along with an improvement of cardiac function.^2^ The safety and therapeutic efficacy was further tested in 3 clinically highly relevant large animal models including ischemic HF initiated by balloon-induced myocardial infarction (MI)^3^^,^^4^ and nonischemic pressure-overload hypertrophy by placing a lumen-reduction stent in the thoracic aorta.^5^ In the early post-MI study with 135 pigs, the treatment was initiated 3 days after the event, repeated on day 28 and a follow-up for 56 days. Two different routes of administration (intracoronary and IV or 2 IV doses) were tested at 3 different dose levels (1, 5, and 10 mg/kg).^3^ In the chronic HF model, administration started at 28 days post-MI followed by 3 or 5 monthly IV dosing of 5 mg/kg that were given until the study end (6 months).^4^ Pigs that underwent nonischemic HF were treated twice at the day of stent implantation as well as 4 weeks later, receiving a cumulative intracoronary dose of 0.5 mg/kg and were followed until day 56.^5^

To assess CDR132L’s efficacy, serial cardiac magnetic resonance, biomarkers, and target engagement markers were evaluated uncovering comparable treatment-related outcomes in all 3 large animal models^3^, ^4^, ^5^: CDR132L cardiac tissue concentrations showed sufficient target tissue exposure, a dose-dependent linear increase, and an inverse reduction of functional cardiac miR-132 level, suggesting a successful target engagement. Histological assessment revealed a significant reduction of cardiac fibrosis and maladaptive cardiomyocyte hypertrophy and an improvement in capillary density. Along with the morphological changes, the drug effectively improved myocardial function indicated by positive changes in left ventricular ejection fraction (LVEF) and beneficial effects on adverse left ventricular remodeling by an increase of the left ventricular end-systolic volume at the end of all follow-up periods. In addition to functional improvements, CDR132L reversed N-terminal prohormone of brain natriuretic peptide (NT-proBNP), a clinically highly relevant HF biomarker.^3^^,^^4^ Finally, the compound was safe and well tolerated at a wide pharmacological range. These studies provided strong efficacy data that, along with an elaborated safety program, paved the way for the approval of the first clinical trial of an microRNA-targeting antisense drug in HF patients (Central Illustration).

In a Phase 1b randomized, placebo-controlled, double-blind, dose-escalation study (NCT04045405) safety and tolerability, pharmacokinetics, target engagement, and exploratory pharmacodynamic effects of CDR132L were assessed in chronic ischemic HF patients receiving standard-of-care therapy. The study enrolled 28 patients with LVEF between 30% and 50% or NT-proBNP >125 ng/L who received a randomly assigned dose between 0.32, 1, 3, and 10 mg/kg body weight or placebo (5:2 in 4 cohorts) by 2 short-term IV infusions (days 1 and 28). During the 128-day study period, CDR132L met all endpoints and showed excellent tolerability and safety. Target engagement was confirmed by a dose-dependent and sustained reduction of plasma miR-132. Further, treatment with CDR132L provoked a clinically meaningful reduction of the median NT-proBNP level, QRS complex narrowing, and an indicative decrease of myocardial fibrosis biomarkers, demonstrating beneficial effects on top of standard of care.^1^ Despite the small number of patients, the pharmacodynamic effects were encouraging as they are in line with the efficacy seen in large animals.^3^, ^4^, ^5^ Therefore, CDR132L recently reached a second milestone in the field of cardiovascular microRNA therapeutics by initiation of HF-REVERT (Study to Assess Efficacy and Safety of CDR132L in Patients With Reduced Left Ventricular Ejection Fraction After Myocardial Infarction), a multicenter Phase 2 study assessing efficacy and safety of CDR132L in 280 patients with reduced LVEF (HFrEF: LVEF ≤45%) early post-MI (NCT05350969). An additional Phase 2 study in a subgroup of HF patients with preserved LVEF will be initiated soon (REMOD-REVERT [Reverse Remodeling Effects of CDR132L in Patients With Heart Failure With Mildly Reduced or Preserved Ejection Fraction and Cardiac Hypertrophy; NCT05953831]).

## A Paradigm Shift in the Treatment of Cardiovascular Diseases Based on Information From the Dark Genome

Small interfering RNA drugs and mRNA vaccines have already reached clinical reality. As a microRNA therapeutic, CDR132L complements this field of new exciting class of drugs. Relying both on a potent ASO chemistry with remarkable target affinity, specificity and safety CDR132L shows outstanding drug qualities including stability and attractive pharmacokinetic properties. CDR132L has a distinctive cardiac target signature through which complex disease-related molecular changes can be reprogrammed, leading to a concerted therapeutic effect in the diseased heart. This approach has the potential to move closer from a symptomatic toward a more etiological approach transforming the treatment of cardiovascular disease affecting millions of patients.

## Funding Support and Author Disclosures

This work was supported by Cardior Pharmaceuticals GmbH, where Dr Thum is founder and shareholder. Dr Viereck is a full-time employee of Cardior Pharmaceuticals GmbH. Dr Thum filed and licensed patents through the Hannover Medical School to Cardior Pharmaceuticals GmbH.
